# Do different rates of gene flow underlie variation in phenotypic and phenological clines in a montane grasshopper community?

**DOI:** 10.1002/ece3.5961

**Published:** 2019-12-30

**Authors:** Rachel A. Slatyer, Sean D. Schoville, César R. Nufio, Lauren B. Buckley

**Affiliations:** ^1^ Department of Entomology University of Wisconsin‐Madison Madison WI USA; ^2^ University of Colorado Natural History Museum University of Colorado Boulder CO USA; ^3^ National Science Foundation Alexandria VA USA; ^4^ Department of Biology University of Washington Seattle WA USA

**Keywords:** climate change, elevational gradient, gene flow, local adaptation, population genetics, Rocky Mountains

## Abstract

Species responses to environmental change are likely to depend on existing genetic and phenotypic variation, as well as evolutionary potential. A key challenge is to determine whether gene flow might facilitate or impede genomic divergence among populations responding to environmental change, and if emergent phenotypic variation is dependent on gene flow rates. A general expectation is that patterns of genetic differentiation in a set of codistributed species reflect differences in dispersal ability. In less dispersive species, we predict greater genetic divergence and reduced gene flow. This could lead to covariation in life‐history traits due to local adaptation, although plasticity or drift could mirror these patterns. We compare genome‐wide patterns of genetic structure in four phenotypically variable grasshopper species along a steep elevation gradient near Boulder, Colorado, and test the hypothesis that genomic differentiation is greater in short‐winged grasshopper species, and statistically associated with variation in growth, reproductive, and physiological traits along this gradient. In addition, we estimate rates of gene flow under competing demographic models, as well as potential gene flow through surveys of phenological overlap among populations within a species. All species exhibit genetic structure along the elevation gradient and limited gene flow. The most pronounced genetic divergence appears in short‐winged (less dispersive) species, which also exhibit less phenological overlap among populations. A high‐elevation population of the most widespread species, *Melanoplus sanguinipes*, appears to be a sink population derived from low elevation populations. While dispersal ability has a clear connection to the genetic structure in different species, genetic distance does not predict growth, reproductive, or physiological trait variation in any species, requiring further investigation to clearly link phenotypic divergence to local adaptation.

## Graphical abstract

Four grasshoppers species exhibit genetic structure and limited gene flow along elevation gradients in the Rocky Mountains. The strength of genetic divergence is associated with dispersal phenotypes and phenological patterns. The most widespread species, *Melanoplus sanguinipes*, follows a stepping stone pattern, with the highest elevation population appearing to be a sink population derived from low elevation.

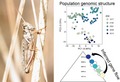

## 1 INTRODUCTION

There is increasing recognition that species' responses to environmental change depend on their potential for plastic and adaptive responses (Hoffmann & Sgrö, 2011), and yet the evolutionary potential of populations remains difficult to determine due to the complex genetic architecture and quantitative nature of key life‐history and physiological traits (Lande, 1982). Steep environmental gradients are a promising context in which to study the evolution of these phenotypic traits, as environmental selection is strong and genetic differences among populations often reflect the forces of selection rather than long periods of genetic isolation (Savolainen, Lascoux, & Merilä, 2013). However, it is well known that life‐history and physiological phenotypes manifest both from plasticity in response to environmental conditions (Angilletta, Steury, & Sears, 2004; Buckley, Nufio, Kirk, & Kingsolver, 2015; Nylin & Gotthard, 1998) and local adaptation associated with genetic differentiation (Dahlhoff et al., 2008; Natarajan et al., 2015). Understanding how gene flow facilitates or impedes genomic divergence among populations, and whether emergent phenotypic variation in life‐history and physiological traits is limited by gene flow rates, remains an important challenge for predicting how populations respond to environmental change.

One classic example of environmentally driven phenotypic clines involves insect species found along elevational gradients, where higher elevation populations exhibit shorter wings, concomitant with a reduction in flight capacity and dispersal distance (Wagner & Liebherr, 1992). Temperature and the length of the active season decrease rapidly with elevation, and these two factors are particularly important for insects, in which rates of growth and development, digestive efficiency, and mortality are tightly linked to the thermal environment (Deutsch et al., 2008; Frazier, Huey, & Berrigan, 2006; Scriber & Lederhouse, 1992). Higher elevation insect populations are often larger than their lower elevation counterparts due to slower development in cooler environments, especially when wings are reduced in size (Angilletta et al., 2004; McCulloch & Waters, 2018). Alternatively, some species (especially fully winged species) reverse this pattern, becoming smaller in order to rapidly complete development during a limited active season (Dearn, 1977; Klok & Harrison, 2013; Shelomi, 2012). A decline in fecundity with elevation is common and is attributed to a decline in body size (for some species) and/or departure from physiological optima (Hodkinson, 2005). Thus, there often appear to be fitness trade‐offs, yet it remains unclear whether these trait differences are due to adaptive phenotypic plasticity or local adaptation. Despite extensive work on a variety of species along elevation gradients, there has been limited investigation of underlying gene flow patterns. Broad surveys of plant and animal studies have shown frequent genetic and phenotypic divergence with altitude (Keller, Alexander, Holderegger, & Edwards, 2013; Ohsawa & Ide, 2008). For example, several studies have shown strong differences in allele frequencies of metabolic enzymes in insect populations (Orr, Porter, Mousseau, & Dingle, 1994; Rank & Dahlhoff, 2002). However, more effort is needed to integrate explicit measurements of gene flow with these well‐studied elevational patterns, in order to understand directionality and rates of gene flow.

The genetic basis of life‐history and physiological divergence has important implications for climate change responses, as phenotypic plasticity allows individuals to track local environmental optima and variation over short timescales, but may ultimately slow evolutionary responses to more pronounced environmental change (Ghalambor et al., 2015; Ghalambor, McKay, Carroll, & Reznick, 2007; Price, Qvarnström, & Irwin, 2003). Local adaptation to different environments could speed up evolutionary responses to climate change and potentially allow greater phenotypic change, but is unlikely to have evolved if populations have high gene flow (Bridle & Vines, 2007; Kremer et al., 2012; Lenormand, 2002; Moritz et al., 2012). At present, it remains difficult and costly to determine genetic adaptation from large insect genomes. Fortunately, patterns of genetic structure and gene flow at neutral markers can be informative about underlying evolutionary processes (Crispo, 2008). As a general expectation, if phenotypic variation along an elevational gradient is driven by local adaptation, as opposed to phenotypic plasticity, one would expect to see evidence of genetic differentiation and limited gene flow, aligned with patterns of phenotypic variation (Kawecki & Ebert, 2004; Sultan & Spencer, 2002). While phenotypic variation might exist simply as a result of genetic drift, it is not expected to covary systematically in relation to an environmental gradient. Reducing gene flow over strong environmental gradients is often required for local adaptation to emerge (Cassel‐Lundhagen, Kaňuch, Low, & Berggren, 2011). This assumption about gene flow generally holds, except when phenotypic traits are controlled by a small number of genes of large effect (Pfeifer et al., 2018; Tigano & Friesen, 2016), as these phenotypes could be maintained by divergent selection despite strong gene flow.

**Figure 1:**
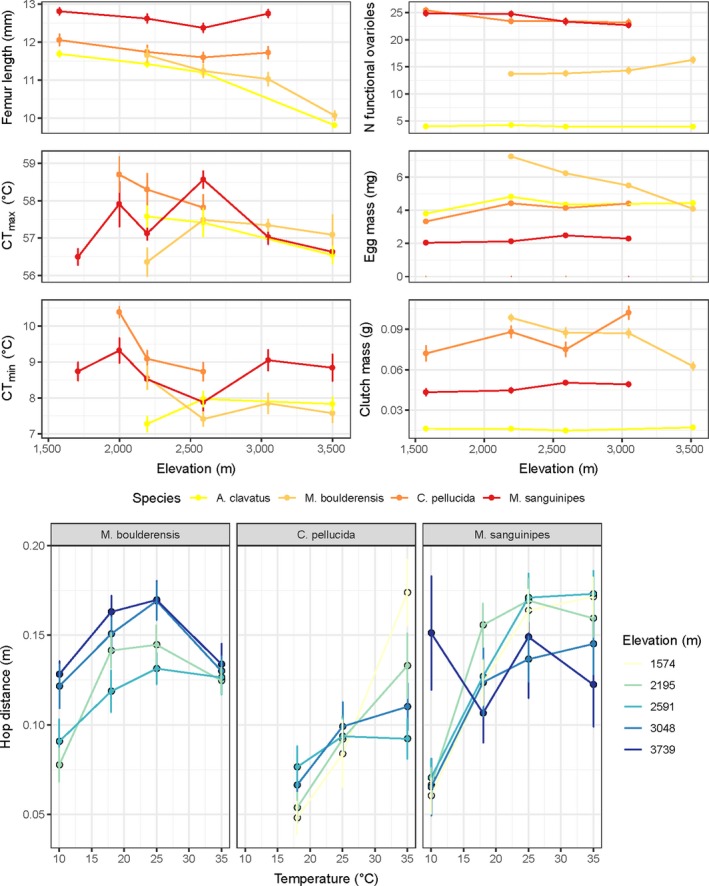
Species vary in the strength and direction of clines for morphological, physiological, and reproductive phenotypes along the elevation gradient (top). Femur length is a proxy for body size and critical thermal minima and maxima (CT_min_ and CT_max_, respectively) indicate thermal constraints on performance. We approximate reproductive potential as the number of functional ovarioles (egg‐producing tubules). We additionally depict egg and clutch (eggs within a pod) mass. Species and populations (color) additionally vary in the temperature dependence of hopping performance (mean distance, bottom). The data suggest that species are (left to right) cool adapted, warm‐adapted, and thermal generalists. We depict means and standard error for field‐collected grasshoppers. Data are previously published (Buckley & Nufio, 2014; Buckley et al., 2014; Levy & Nufio, 2015)

Here, we test the hypothesis that clinal differences in growth and reproduction “(life‐history traits)” and physiological traits of four ecologically divergent grasshopper species (Buckley & Nufio, 2014; Buckley, Nufio, & Kingsolver, 2014; Levy & Nufio, 2015) are associated with genetic differentiation and reduced demographic estimates of gene flow (local adaptation). We examine a large set of genome‐wide single nucleotide polymorphisms for each species to estimate genetic parameters. Our study focuses on a steep environmental gradient near Boulder, Colorado, spanning 2,000 m elevation over a horizontal distance of ~35 km, where *Melanoplus boulderensis* and *Aeropedellus clavatus*, short‐winged, early‐season species, can be compared to *Camnula pellucida* and *Melanoplus sanguinipes*, long‐winged, mid to late‐season species. Short‐winged and flightless grasshopper species should have reduced dispersal potential, show greater genetic differentiation among geographical populations and a greater potential for local adaptation than long‐winged species (Knowles, 2000; Ortego, García‐Navas, Noguerales, & Cordero, 2015; Tinnert & Forsman, 2017). The two long‐winged species are considered more dispersive, because in addition to occurring at a broader range in elevation, they are occasionally collected as “accidentals” (nonresident migrants) at high‐elevation sites along the gradient (Alexander, 1964). Phenotypic clines in morphological, physiological, and reproductive traits have been well documented among these species (Table 1, Figure 1), with notable differences among our four focal taxa corresponding to ecological divergence (Buckley & Nufio, 2014; Buckley et al., 2014; Levy & Nufio, 2015).

**Table 1:** Sample sites and characteristics of grasshopper species (see map Figure 2)

Elevation (m a.s.l.)	Latitude (°N)	Longitude (°E)	Life zone classification	Mean annual temperature (°C)	Mean season length (days)	Species collected
1,577 (RF/CM)	40.061	−105.193	High plains	10.67	199.8	ac, ms*
2,195 (A1)	40.015	−105.377	Lower montane	7.93	164.5	ac, mb, ms, cp
2,591 (B1)	40.023	−105.430	Upper montane	5.98	148.0	ac, mb, ms, cp
3,048 (C1)	40.036	−105.547	Subalpine	1.79	100.5	mb, ms, cp
3,515 (D1)	40.059	−105.617	Alpine	−3.50	84.4	ac, mb, ms

Species	Dispersive	Seasonal timing	Size	TPC adaptation	CT_min_, CT_max_	Metabolic rate	*N* functional ovarioles	Egg mass	Clutch mass
*Melanoplus boulderensis*	No	Early	↓	Cool	Low	↑	↑	↓	↓
*Aeropedellus clavatus*	No	Early	↓	?	?	≈	≈	≈	≈
*Camnula pellucida*	Yes	Middle	≈	Warm	High	≈	↓	↑	↑
*Melanoplus sanguinipes*	Yes	Late	≈	Generalist	High	↑	↓	↑	↑

Note

* Arrows indicate the relative increase (up/down) in each trait with elevation. Species names are abbreviated as follows: *A. clavatus* (ac), *M. boulderensis* (mb), *Melanoplus sanguinipes* (ms), and *C. pellucida* (cp). The populations at ~1,577 m are from Red Fox (RF: *A. clavatus*) and Chautauqua Mesa (CM: *M. sanguinipes*) as in Levy and Nufio (2015).

While phenotypic variation is evident in all four grasshopper species, it is unclear how that variation is related to geographical, environmental, and genetic factors. We first compare population genetic structure and test for an association between genetic divergence and observed phenotypic clines. Ideally, such a comparison would be based on the additive genetic variation in phenotypic traits, but lacking such data for each population, we focus on correlations between phenotypic traits and geographical, environmental and genetic variables. Because genetic differentiation can emerge even with substantial gene flow (such as in a source‐sink model or with strongly asymmetrical gene flow: Gaggiotti, 1996; Lowe & Allendorf, 2010), we next develop models varying the demographic processes that underlie population genetic patterns. We employ model selection approaches to compare these competing spatial models of gene flow for each grasshopper species. We then extend this analysis to investigate whether an ecological determinant of gene flow, phenological overlap among populations, is consistent with our observed demographic estimates. Phenological mismatch has been observed to directly reduce gene flow across elevation gradients, particularly in the downslope direction (Peterson, 1997). Limited temporal overlap among populations along an elevational gradient would reduce gene flow and increase genetic structure. Our previous research has shown that phenology in these grasshopper species is temperature dependent and occurs earlier in warmer years (Nufio & Buckley, 2019). Our hypothesis is that the short‐winged species will exhibit genetic differentiation, limited phenological overlap, and gene flow, and have a strong association between genetic divergence and phenotypic differentiation along the elevational cline. Conversely, long‐winged species might exhibit limited genetic differentiation, a source‐sink pattern of gene flow, high phenological overlap, and have no association of genetic divergence with phenotypic differentiation.

## 2 METHODS

### 2.1 Study area, species, and phenotypic variation

**Figure 2:**
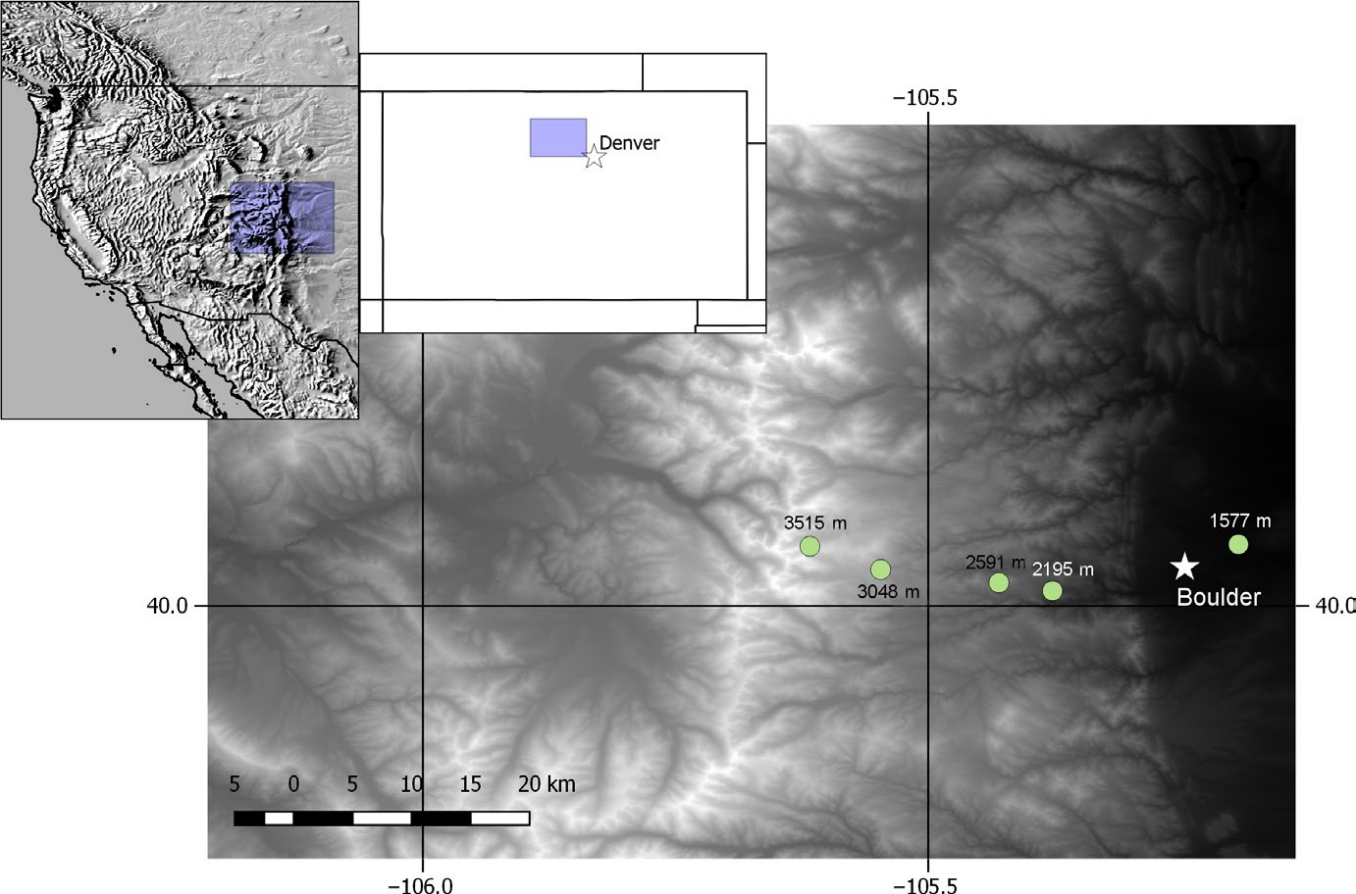
Map of the study region and sample sites near Boulder, Colorado, USA

We examine grasshopper populations spanning an elevation gradient of 1,500–3,500 m along the 40th N parallel in Boulder County, Colorado, USA (Table 1, Figure 2). The sites are all grassy meadows, with somewhat denser vegetation at the lower elevation sites. The forest matrix at the montane sites is less continuous and has been more impacted by forest fires than the subalpine site. The alpine site is an open ridgeline, but separated from the subalpine site by continuous forest vegetation (aspens and pines). All four of the species occur in montane foothill habitat up to subalpine habitat, while *M. boulderensis* (part of the *Melanoplus dodgei* species complex, (Otte, 2012), *A. clavatus* and occasionally *M. sanguinipes* occur in alpine habitats. *Melanoplus boulderensis* is a neoendemic of the Rocky Mountains, likely having evolved in response to glacial cycles over the last million years (Knowles & Otte, 2000), while the other three species are widespread in North America. The grasshoppers eat a variety of forbs and grasses with the exception of the grass specialist *A. clavatus*. The species overwinter in an egg diapause and are univoltine (annual) with the exception of *A. clavatus*, which takes 2 years to develop at higher elevations (Alexander & Hilliard, 1964).

These populations were studied by Gordon Alexander et al. in the late 1950's. A resurvey program between 2006 and 2015 showed that populations and species have shifted their phenologies and relative abundances over the last 50 years (Nufio & Buckley, 2019; Nufio, McGuire, Bowers, & Guralnick, 2010), concurrent with climate warming during this period (McGuire, Nufio, Bowers, & Guralnick, 2012). Phenological timing has advanced most dramatically at high‐elevation sites, where warming has been greater. Each species and their local populations along the elevational gradient differ in a number of phenotypic traits (Figure 1). The short‐winged species *M. boulderensis* and *A. clavatus* decrease in body size with elevation (a growth trait), whereas the long‐wing species exhibit no cline in body size. Reproductive traits, including ovariole number and resource allocation to eggs, vary among populations along the gradient, and in different directions for long‐winged and short‐winged species. Physiological traits related to temperature tolerance and performance also vary among populations. Clinal differences in thermal tolerances (critical thermal minima and maxima) and preferences (preferred body temperature) are greater for the long‐winged species (*C. pellucida* and *M. sanguinipes*) than the short‐winged species, while these species also exhibit higher thermal limits (Buckley et al., 2014). All species increase feeding and digestion rates with temperature, but the temperature dependence of performance (hopping distance) varies among species (Buckley & Nufio, 2014). Metabolic rates increase with elevation for *M. boulderensis* and *M. sanguinipes* (in regressions accounting for mass and temperature), but do not vary with elevation for the other species (Table 1). However, laboratory‐based rearing experiments have suggested that some physiological traits in *M. sanguinipes* vary as a result of phenotypic plasticity (Buckley & Nufio, 2014; Buckley et al., 2015). Furthermore, phenological patterns are known to be related to developmental plasticity, as the early‐season species *M. boulderensis* and *C. pellucida* slow their development rate (in terms of physiological time) at the high‐elevation site in response to warming (Buckley et al., 2015).

### 2.2 Genetic sampling, DNA extraction, and genotype‐by‐sequencing

Grasshoppers were collected from the field in June and August 2016, from three to five different locations, depending on species (Table 1). Twenty adults of each species were collected from each location, where possible, with approximately even numbers of males and females. Specimens were frozen then transferred to 100% ethanol for storage prior to DNA extraction. DNA was extracted from hind femur muscle tissue using a Qiagen DNeasy^®^ 96 Blood & Tissue Kit (Qiagen). We followed the manufacturer's protocol with one modification: 4 μl RNase A (100 mg/ml) was added after the initial lysis step. DNA was eluted in 2 × 100 μl of the supplied elution buffer. DNA concentrations were quantified with a Qubit^®^ 2.0 Fluorometer using a dsDNA High Sensitivity Assay Kit (Life Technologies). At least 300 ng of DNA per individual was sent to the University of Wisconsin‐Madison Biotechnology Center for library preparation and sequencing using a double‐digest genotype‐by‐sequencing (GBS) protocol (Elshire et al., 2011). In brief, DNA was digested with the restriction enzymes Msp1 and Pst1, Illumina adapters and unique barcode adapters were ligated to the digested fragments, and fragments were pooled across individuals and amplified with the Illumina Solexa PCR protocol. For each species, samples and a no‐template control were prepared in one GBS library. Each library was sequenced separately on a single Illumina HiSeq 2000 (Illumina) lane using single‐end 100‐bp reads. Raw Illumina reads were deposited in the National Center for Biotechnology Information Short Read Archive (accession no. SRR9216863–SRR9216866) under Bioproject PRJNA547722.

### 2.3 Read processing and SNP ascertainment

Raw reads were split by individual barcode using the *process_radtags* script in stacks v1.44 (Catchen, Hohenlohe, Bassham, Amores, & Cresko, 2013). Read quality and adapter contamination were screened with fastqc (Babraham Bioinformatics, 2011). Barcodes at the 5′ end were trimmed with trimmomatic v0.36 (Bolger, Lohse, & Usadel, 2014) to remove adapter sequences and poor base calls. Single nucleotide polymorphisms (SNPs) were called from aligned reads within stacks. stacks parameter values consisted of a minimum stack depth of 8 to report a locus (m parameter), two mismatches allowed between loci when building the SNP catalog (n parameter), and the presence of the SNP in at least two of the focal populations (p parameter).

In order to infer missing genotype calls, the vcf file for each SNP dataset was processed in Fastphase v1.4 (Scheet & Stephens, 2006), which was developed to impute genotypes given the observed allele frequencies and linkage among SNPs in each subpopulation. The number of random starts of the expectation‐maximization algorithm was set to 10, and the program was run without haplotype estimation. Missing genotypes were estimated for each sample site within each species, and those genotypes with uncertainty (<70% probability) were left as missing data. SNPs were then filtered by removing loci that had an overall minor allele frequency of <2.0%. After this step, no loci or individuals contained missing data.

### 2.4 Population genetic analyses

For each SNP we calculated the following quantities: minor allele frequency (MAF), observed (*H*_O_) and expected (*H*_E_) heterozygosity, within‐population gene diversity (*H*_S_), and Wright's inbreeding coefficient (*F*_IS_). Each statistic was averaged across loci within populations, and also calculated overall for each species. Additionally, for each species, we calculated overall gene diversity (multilocus heterozygosity; *H*_T_) and *D*_est_ (Jost, 2008). These measures are useful for assessing the overall genetic diversity found within and among species. To provide quantitative estimates of genetic differentiation among populations, we estimated the fixation index (*F*_ST_) in a pairwise manner using Nei's *D* (Nei, 1978) and a linearized version based on Latter's equation (Latter, 1972). All summary statistics were calculated using the “basic.stats” function in the hierfstat package (Goudet, 2005; Goudet & Jombart, 2015) in r v3.4.0 (R Core Team, 2017), with modifications for biallelic SNP data (custom R script for these calculations are available on DRYAD: https://doi.org/10.5061/dryad.w3r2280m3). Pairwise *F*_ST_ between populations within a species was computed in hierfstat using the “pairwise.neifst” function. Additionally, we tested for association of genetic divergence with geographical distance (isolation by distance) using the Mantel test function in the vegan package in R (Oksanen et al., 2013). Mantel tests were used to test the Pearson product‐moment correlation of a genetic distance matrix based on linearized *F*_ST_ to Euclidean geographical distance, with 999 permutations to test for significance.

As a first step in exploring population structure, we used a principal components analysis (PCA) in R, on centered and scaled SNP datasets for each species. Second, we examined genetic admixture using snmf (Frichot, Mathieu, Trouillon, Bouchard, & François, 2014), implemented using the “snmf” function in LEA 1.6.0 package (Frichot & François, 2015) in R. For each species, we conducted 10 independent runs at *K* = 2 to *K* = *N*, where *K* is the number of ancestral populations and *N* is the total number of populations sampled, and combined the results from each *K* using CLUMPP (Jakobsson & Rosenberg, 2007), via the “clumppExport” function in pophelper v2.0.0 (Francis, 2017) in R. CLUMPP averages the individual admixture coefficients from each run and these results were visualized using pophelper. As an alternative clustering method, we used the program admixture 1.3.0 (Alexander, Novembre, & Lange, 2009), which implements a maximum likelihood approach to estimating ancestry. We used a fivefold cross‐validation procedure to identify the value of *K* (the number of ancestral populations) for which the model has best predictive accuracy, testing *K* = 1 to *K* = *N* where *N* is the total number of populations sampled. We conducted 10 independent runs at each value of *K* and compared the average cross‐validation error. Data conversions were done using custom R scripts and plink 1.9 (Chang et al., 2015).

### 2.5 Correlations with phenotypic clines

Previously published phenotypic data include multiple variables representing growth, reproductive and physiological traits. Growth traits include body mass and femur length, which is correlated with body size. Reproductive traits include clutch size, ovariole number, egg mass, and egg clutch weight. Physiological traits include preferred body temperature, critical thermal minima, and critical thermal maxima. For each trait type, variables were combined to compute the phenotypic distance among populations based on mean values. We then tested for phenotypic correlations with the following predictor variables: geographical distance, elevation, environmental distance (based on a composite of mean annual temperature and the mean growing season length in days), and genetic distance (the linear estimate of pairwise *F*_ST_). We used the vegan package in R to compute dissimilarity matrices using Euclidean distance, and we conducted matrix correlation tests for each species. Mantel tests were used to test the Pearson product‐moment correlation of phenotypic variation to each predictor variable independently, using 999 permutations to test for significance. These tests assume a linear relationship between the two variables and a constant scalar relationship among populations.

### 2.6 Modelling rates and direction of gene flow

We set out to test alternative models of gene flow among populations and estimate gene flow rates. We employed an Approximate Bayesian computation (ABC) approach (Beaumont, Zhang, & Balding, 2002) to fit our data to a set of four spatially explicit models for each species (Figure S1): (a) unidirectional gene flow downslope, (b) unidirectional gene flow upslope, (c) bidirectional gene flow in a stepping stone model, and (d) equal and constant gene flow among all populations (island model). Alexander (1964) suggested that *M. sanguinipes* occurs as an “accidental” (i.e., sink population) at elevations above ~3,000 m a.s.l, with adults sourced from lowland populations. Therefore, for this species, we additionally tested a source‐sink model where all populations formed an island model and could contribute unidirectional gene flow to the upper elevation population D1. The ABC method estimates the posterior distribution of model parameters without calculating the explicit likelihood of these parameters. Instead, data are simulated under each model by randomly drawing values from a prior distribution of parameter values, the observed data are compared to synthetic data based on similarity to summary statistic values, and both parameters and models can be quantitatively compared using posterior distributions and Bayes Factor (BF) scores (Csilléry, Blum, & Francois, 2012). We used the coalescent simulator fastsimcoal2 v2.5 (Excoffier, Dupanloup, Huerta‐Sánchez, Sousa, & Foll, 2013) to generate two million synthetic data sets per model, per species (input files are available on DRYAD: https://doi.org/10.5061/dryad.w3r2280m3). The synthetic datasets had population sample sizes matched to the relevant species, migration rate (proportion of individuals in the population per generation) drawn from a uniform prior distribution (0.001–0.05), and effective population (*N*_e_) size drawn from a log‐uniform prior distribution (200–10,000). For each model, we simulated two million datasets each with 500,000 independent sequences of 10 base pairs, and drew SNPs in equal number to those available in the empirical dataset for each species. We then used arlsumstat in arlequin v3.5 (Excoffier & Lischer, 2010) to calculate population genetic summary statistics for each simulated dataset, as well as the SNP datasets for each species. These statistics included population‐ and species‐level estimates of diversity (total heterozygosity and segregating substitutions, segregating substitutions per population) and differentiation (number of pairwise differences per populations and pairwise *F*_ST_).

ABC analysis was completed using the abc package (Csilléry et al., 2012) in R. First, we used a leave‐one‐out cross‐validation to determine whether the applied ABC approach should, in principle, be able to distinguish between the proposed models. We used a cross‐validation sample of 100 and tolerance levels of .001, .005, and .01 (accepting the 2,000, 10,000, or 20,000 of closest estimates for each model, respectively), with posterior model probabilities estimated using both a neural network (Blum & François, 2010) and a rejection method (Beaumont et al., 2002). Second, we calculated the posterior probabilities of all model scenarios using rejection and a tolerance value of .001 and selected the top three models for estimating posterior model probabilities using a neural network. For the model with the highest posterior probability, goodness‐of‐fit to the observed values was tested using the “gfit” function in abc with a tolerance of .01 and 1,000 replicates used to estimate the null distribution of the goodness‐of‐fit statistic (median). If two models had high posterior probabilities, goodness‐of‐fit to the observed values was tested for both. The rejection algorithm favors those models which simulate data closest to the observed data, while the neural network attempts to correct for deviation from the simulated data and the observed data. Given the finite number of simulations we employed and the vast state space of the models, we focus on the results from the neural network to provide the most accurate estimate of model parameters. Parameters (population size and gene flow) were estimated under the best fitting model using a tolerance value of .05.

To further investigate parameter estimates and examine the effect of our prior in the ABC simulations, we employed the use of the minor allele site frequency spectrum (AFS) for an independent estimate of parameters under the best fitting model, which can be estimated jointly over multiple populations (Gutenkunst, Hernandez, Williamson, & Bustamante, 2009). We used a maximization procedure for parameter inference from the joint AFS implemented in fastsimcoal2 v2.5 (Excoffier et al., 2013). We chose the following sampling criteria for maximization: between 100,000 (‐n parameter) and 250,000 (‐N parameter) simulations were run, parameters values were estimated following 10 to 40 ECM cycles (‐l and ‐L parameters, respectively), and we allowed maximization to stop when we observed 0.001 minimum relative difference (maximum composite likelihood, or ‐M parameter) in parameters between two iterations of the run. We employed the same prior distributions for population genetic parameters as employed in the ABC analysis. We repeated this 50 times for each species to get the parameter combination with the highest maximum likelihood across all runs. Confidence intervals around these estimates were generated using parametric bootstrap with 1,000 replicates in fastsimcoal2 and R.

### 2.7 Phenological differences among sites

To provide an ecological measure of gene flow potential between populations, we measured the degree of phenological overlap. Weekly survey data from 1959 to 1960 were assembled from field notebooks as part of the Gordon Alexander Project (http://ghopclimate.colorado.edu). Weekly resurveys were conducted 2006–2015 (Nufio et al., 2010) following the original protocol, consisting of 1.5 person‐hour of sweep netting (divided among 1–3 surveyors) and 0.75 person‐hours of searching for adults and juveniles that may have been missed by sweep netting (Alexander & Hilliard, 1969). We assessed phenological overlap between sites for each species using Pianka's symmetric metric of niche overlap, which ranges from 0 to 1 (no to complete overlap) (Fleming & Partridge, 1984). The metric accounts for the proportion of individuals that occur during each sampling period at each site and sums across the survey season. Because sites were sometime sampled on different days within a week, we grouped the sampling period by week. We additionally quantified phenological overlap by calculating the day of year that the 15th and 85th percentile adult was surveyed for each site and species within each season.

## 3 RESULTS

### 3.1 Diversity and population genetic structure

A total of 18,604 to 29,213 SNPs were identified in the four species (Table 2). After data imputation and filtering for MAF >2%, the final data set included 4,087 to 9,454 SNP loci, genotyped in 65–96 individuals per species (Table 2). Genetic diversity varied little between populations and species, with the exception of the 3,048 m population of *M. boulderensis*, which had considerably lower gene diversity and heterozygosity than the other populations (Table 2). Levels of inbreeding were slightly higher for *M. sanguinipes* than the other three species, but were similar across populations. While population genetic differentiation was low to moderate for all species, the two long‐winged species, *M. sanguinipes* (*F*_ST_ = 0.047) and *C. pellucida* (*F*_ST_ = 0.037)*,* showed less differentiation than the two short‐winged species, *M. boulderensis* (*F*_ST_ = 0.081) and *A. clavatus* (*F*_ST_ = 0.062) (Table 2). Pairwise *F*_ST_ among populations was low to moderate for all species (Table 3), although *M. boulderensis* at 3,048 m showed particularly high differentiation compared to all other populations, perhaps due to its reduced genetic diversity. Notably, almost no genetic differentiation was found between the lowest (1,577 m) and highest (3,515 m) populations in *M. sanguinipes*. Mantel tests showed no association with geographical distance for *M. boulderensis* (*r* = −.276, *p* = .71), *M. sanguinipes* (*r* = −.576, *p* = .88), and *C. pellucida* (*r* = .525, *p* = .33), but isolation by distance was significant for *A. clavatus* (*r* = .673, *p* = .042).

**Table 2:** Summary statistics for filtered SNP loci

Species and population	*N*	*T*	MAF	SNP	*H*_O_	*H*_E_	*H*_S_	*F*_IS_	*F*_ST_	*H*_T_	*D*_est_
*Aeropedellus clavatus*	87	29,213	0.13	9,454	0.167	0.202	0.193	0.138	0.047	0.203	0.016
1,577 m	22			6,811	0.161	0.184	0.189	0.089			
2,195 m	21			6,834	0.17	0.185	0.19	0.062			
2,591 m	22			7,316	0.174	0.197	0.202	0.085			
3,515 m	22			6,815	0.161	0.187	0.192	0.102			
*Melanoplus boulderensis*	66	23,219	0.09	5,501	0.116	0.149	0.132	0.123	0.062	0.141	0.013
2,195 m	21			3,908	0.137	0.149	0.153	0.043			
2,591 m	18			3,548	0.124	0.138	0.143	0.059			
3,048 m	8			1,348	0.063	0.066	0.071	0.06			
3,515 m	19			3,758	0.14	0.156	0.16	0.056			
*Melanoplus sanguinipes*	96	24,376	0.47	4,087	0.163	0.123	0.159	0.224	0.036	0.165	0.009
1,577 m	22			3,322	0.152	0.117	0.156	0.14			
2,195 m	20			2,674	0.132	0.108	0.136	0.095			
2,591 m	21			3,018	0.161	0.133	0.166	0.099			
3,048 m	21			3,135	0.162	0.127	0.167	0.133			
3,515 m	12			2,734	0.16	0.131	0.168	0.125			
*Camnula pellucida*	65	18,604	0.13	6,815	0.162	0.193	0.19	0.149	0.025	0.195	0.009
2,195 m	23			4,615	0.16	0.181	0.186	0.071			
2,591 m	21			4,487	0.164	0.188	0.194	0.085			
3,048 m	21			4,318	0.161	0.185	0.19	0.081			

Note

*N* is the number of analyzed individuals; *T* is the number of GBS loci; SNP is the total number of polymorphic SNPs per species (species rows) or the number of polymorphic SNPs in a population (population rows); MAF is the mean frequency of the less common allele, after filtering; *H*_O_ is the observed heterozygosity, averaged over loci; *H*_E_ is the expected heterozygosity, averaged across loci; *H*_S_ is the within‐population gene diversity; *F*_IS_ is Wright's inbreeding coefficient; *H*_T_ is the multilocus heterozygosity; *F*_ST_ is the fixation index; and *D*_est_ is Jost's population differentiation index.

**Table 3:** Genetic divergence (Nei's pairwise *F*_ST_) among populations of each grasshopper species

*Aeropedellus clavatus*					*Melanoplus sanguinipes*					
	1,577 m	2,195 m	2,591 m	3,515 m		1,577 m	2,195 m	2,591 m	3,048 m	3,515 m
1,577 m	.				1,577 m	.				
2,195 m	0.056	.			2,195 m	0.033	.			
2,591 m	0.056	0.042	.		2,591 m	0.043	0.069	.		
3,515 m	0.074	0.076	0.066	.	3,048 m	0.036	0.060	0.046	.	
					3,515 m	0.005	0.065	0.054	0.042	.

*Melanoplus boulderensis*					*Camnula pellucida*			
	2,195 m	2,591 m	3,048 m	3,515 m		2,195 m	2,591 m	3,048 m
2,195 m	.				2,195 m	.		
2,591 m	0.046	.			2,591 m	0.033	.	
3,048 m	0.120	0.114	.		3,048 m	0.039	0.040	.
3,515 m	0.050	0.058	0.128	.				

Note

All values were significantly different at the *α* = .05 level.

**Figure 3:**
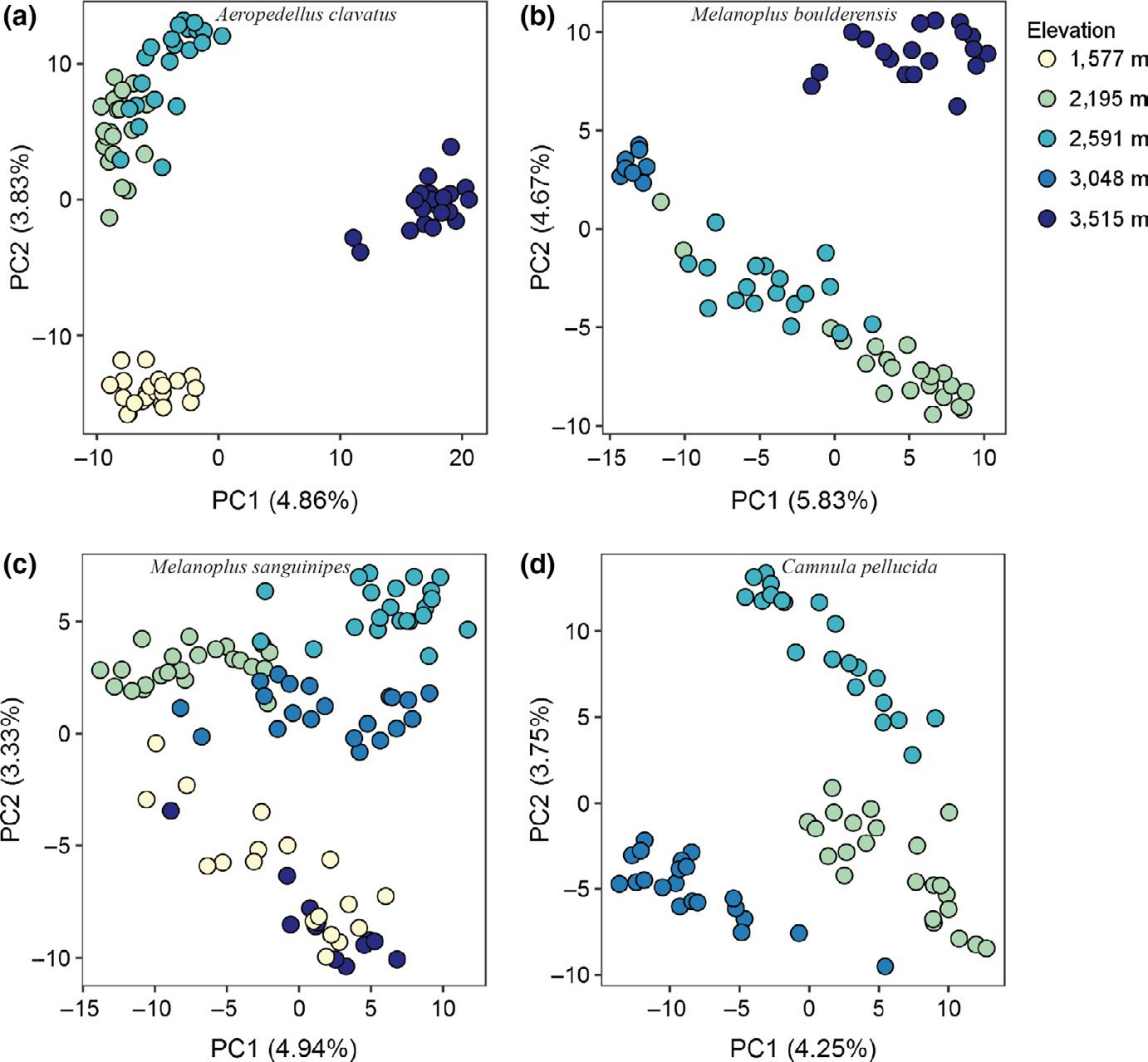
Principal components analysis of filtered SNPS for (a) *Aeropedellus clavatus* (*n* SNPs = 9,454, *n* individuals = 87), (b) *Melanoplus boulderensis* (*n* SNPs = 5,501, *n* individuals = 66), (c) *Melanoplus sanguinipes* (*n* SNPs = 4,087, *n* individuals = 96), and (d) *Camnula pellucida* (*n* SNPs = 5,538, *n* individuals = 65). For each species, individuals were sampled from three to five populations. Darker colors are higher elevations

**Figure 4:**
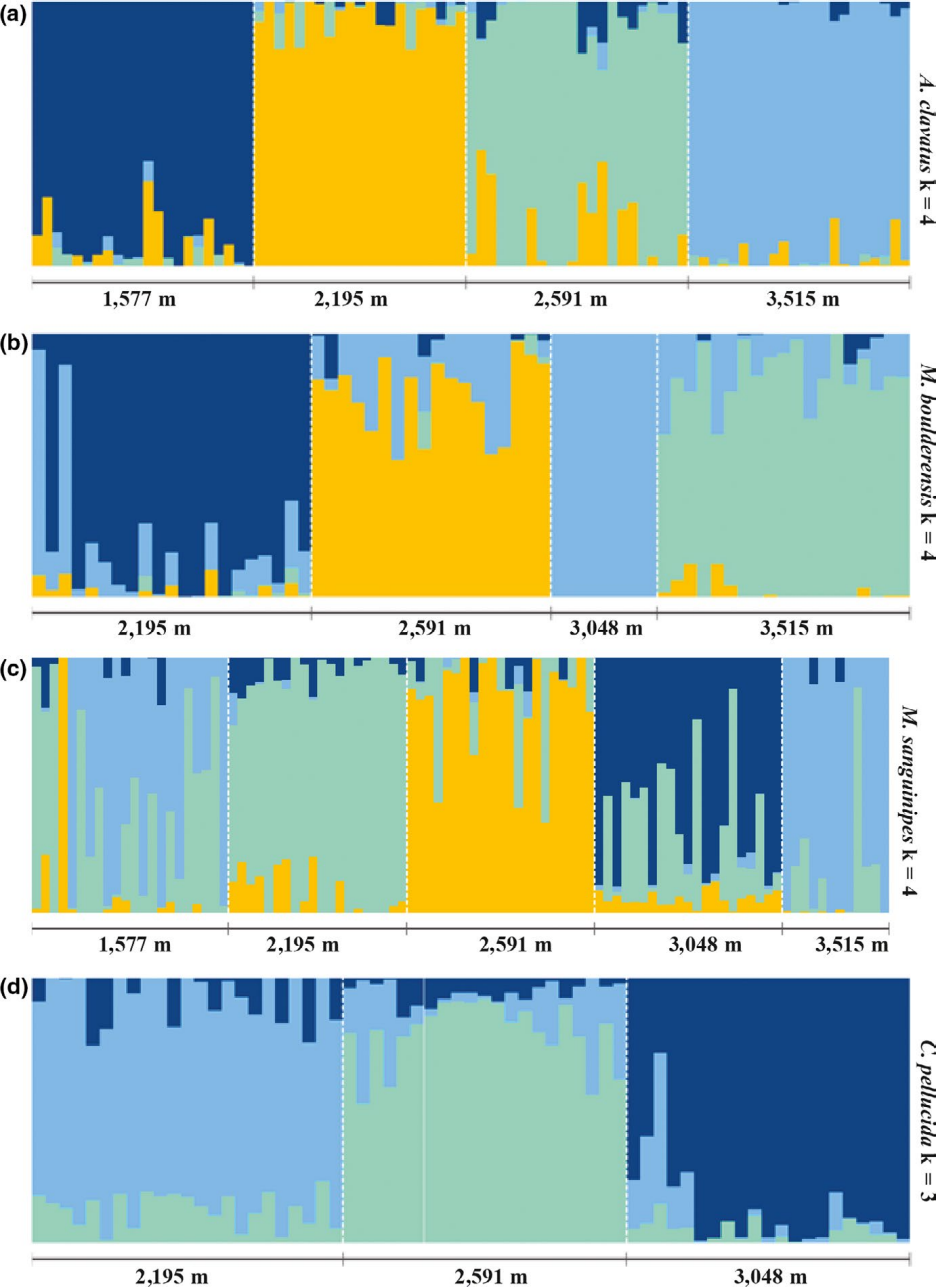
Admixture coefficients for (a) *Aeropedellus clavatus*, (b) *Melanoplus boulderensis*, (c) *Melanoplus sanguinipes*, and (d) *Camnula pellucida*; generated from 10 independent runs with the number of ancestral clusters (*K*) equal to the number of populations sampled, using sNMF. Each bar is an individual and admixture coefficients represent the estimated proportion of an individual's genome originating in each cluster (represented by different colors)

The first two principal components combined explained 8.0% (*C. pellucida*) to 10.5% (*M. boulderensis*) of the genetic variation (Figure 3). All individuals across species are clearly clustered by sample site, with slightly more overlap in mid‐elevation populations. Notably, the clustering patterns for *M. sanguinipes* suggest low differentiation between the lowest and highest populations. Estimates of population structure from the cluster based algorithms sNMF and admixture differed from each other, and from the PCA. The cross‐validation procedure in sNMF (Figure S2) indicates that optimal *K* = 1 for *C. pellucida*, *K* = 2 for *M. sanguinipes*, and *K* = 4 for both *M. boulderensis* and *A. clavatus*. The cross‐validation procedure in admixture (Figure S3) suggests *K* = 1 for all species, although the error increases only slightly with larger *K* values. Considering the overlap among methods, we focus on the sNMF results with *K* = 4 for *M. boulderensis*, *M. sanguinipes*, and *A. clavatus*, and *K* = 3 for *C. pellucida* (Figure 4, but see Figures S4 and S5 for all *K* values for each species under each model). There is clear population structure for all populations and species. Comparing species, *A. clavatus* had the lowest amount of overall admixture, with individuals having, on average, 90.5 ± 13.1% assignment to a single ancestral population. *Melanoplus sanguinipes* had the highest levels of admixture overall, with an average of 75.5 ± 15.0% assignment to a single ancestral population. *Camnula pellucida* and *M. boulderensis* had similar levels of admixture (mean 82.5 ± 11.8% and 84.2 ± 13.8%, assignment to a single ancestral population, respectively).

### 3.2 Association between phenotypic clines and predictor variables

We examined the correlation of phenotypic differences among populations on the elevational gradient and a set of predictor variables (geographical distance, elevational distance, environmental distance, and genetic distance) using Mantel tests and assuming a linear relationship (Table 4). Notably, only short‐winged species had significant phenotypic associations, but not with genetic distance as a predictor variable. Significant (*α* = .05) tests were found in *M. boulderensis* for both growth (*geographic distance r* = .656, *p* = .04; *elevation distance r* = .800, *p* = .04; *environmental distance r* = .481, *p* = .04) and nearly significant (*α* = .10) tests were found for reproductive traits (*geographic distance r* = .853, *p* = .08; *elevation distance r* = .867, *p* = .08; *environmental distance r* = .863, *p* = .08). Significant (*α* = .05) tests were found in *A. clavatus* for growth traits (*geographic distance r* = .642, *p* = .04; *elevation distance r* = .786, *p* = .04; *environmental distance r* = .482, *p* = .04), and growth traits were nearly significant in association with genetic distance (*r* = .889, *p* = .08).

**Table 4:** Mantel tests of geographical, elevational, environmental, and genetic distance as correlates of phenotypic distance

Mantel tests	*Melanoplus sanguinipes*		*Melanoplus boulderensis*		*Aeropedellus clavatus*		*Camnula pellucida*	
	*r*	*p*‐Value	*r*	*p*‐Value	*r*	*p*‐Value	*r*	*p*‐Value
Physiological traits								
*ρ*_phenotype, geography_	−.217	.68	.029	.42	.926	.17	N/A	N/A
*ρ*_phenotype, elevation_	−.240	.68	.185	.33	.852	.13	N/A	N/A
*ρ*_phenotype, environment_	−.034	.51	−.145	.46	.935	.13	N/A	N/A
*ρ*_phenotype, genetic_	.290	.3	−.195	.58	.952	.29	N/A	N/A
Growth traits								
*ρ*_phenotype, geography_	−.337	.76	**.656**	**.04**	**.803**	**.04**	−.906	1
*ρ*_phenotype, elevation_	−.429	.87	**.800**	**.04**	**.927**	**.04**	−.99	1
*ρ*_phenotype, environment_	−.259	.76	**.481**	**.04**	**.964**	**.04**	−.847	1
*ρ*_phenotype, genetic_	−.062	.53	−.377	.58	**.889**	**.08**	−.114	.67
Reproductive traits								
*ρ*_phenotype, geography_	−.870	1	**.853**	**.08**	−.012	.42	.667	.33
*ρ*_phenotype, elevation_	−.821	.98	**.867**	**.08**	−.220	.71	.275	.5
*ρ*_phenotype, environment_	−.720	.975	**.863**	**.08**	−.231	.75	.753	.33
*ρ*_phenotype, genetic_	.691	.12	−.786	.71	−.046	.5	.984	.17

Note

Significant (*α* = .05) or nearly significant (*α* = .10) tests are shown in bold type. Tests labelled as not applicable (N/A) had too much missing data.

Significant tests are shown in bold type.

### 3.3 Modelling gene flow

We compared four competing models of spatial gene flow for each species using ABC methods. For all species, cross‐validation indicated that each model could be distinguished, with a high probability of correctly assigning test samples to a model (rejection method, mean = 95% and 94.5% for tolerance of .001 and .005, respectively; neural network method, mean = 83% and 79% for tolerance of .001 and .005, respectively; see Tables S1–S4). Model selection using a rejection algorithm and neural network suggested a single best candidate model for *A. clavatus*, the upslope gene flow model (Table 5). However, rejection and neural network approaches favored alternative models for the other three species. In all three cases, downslope directional gene flow was favored by the rejection algorithm, whereas posterior probabilities from the neural network favored the stepping stone model with bidirectional gene flow. In *M. boulderensis*, goodness‐of‐fit tests indicated significant differences between the simulated distribution of genetic data in the downslope gene flow model and observed values, thereby rejecting this model (Table 5). However, the goodness‐of‐fit tests did not reject the alternative models for *C. pellucida* or *M. sanguinipes*, and in the case of *M. sanguinipes* it indicated a poor fit of the summary statistics to the best fitting stepping stone model. As a result, the stepping stone, downslope gene flow, and source‐sink models were used for parameter estimation in *M. sanguinipes*.

**Table 5:** Results of model selection using a tolerance of .001 and two methods (rejection, neural network), with best model shown in bold type

Species	Model	Proportion of accepted simulation (rejection method)	Posterior model probabilities (neural network)	Goodness‐of‐fit (*p*‐value)
*Melanoplus boulderensis*	dn	**.9099**	.3355	.02
	up	.0898	.0436	.011
	ss*	.0004	**.6209**	.104
	am	.00	–	–
*Aeropedellus clavatus*	dn	.071	.006	.44
	up*	**.929**	**.994**	.44
	ss	.0	–	–
	am	.0	–	–
*Melanoplus sanguinipes*	dn*	**.9294**	.0458	.364
	up	.0135	.0195	–
	ss*	.0023	**.6876**	.036
	am	.0004	.0488	–
	sk*	.0544	.1984	.325
*Camnula pellucida*	dn	**.7773**	.1170	.134
	up	.1168	.1126	.062
	ss*	.0594	**.7104**	.444
	am	.0465	–	–

Note

Models refer to—dn: unidirectional downslope gene flow; up: unidirectional upslope gene flow; ss: bidirectional stepping stone gene flow; am: equal rates of gene flow in an island model; sk: source‐sink gene flow. For some species, not all models could be tested using the neural network method because infinite values were produced for lower likelihood models. Therefore, the top two or three models were compared in the neural network, and the model(s) with the highest posterior probability were used for downstream parameter estimation (indicated by *). Goodness‐of‐fit tests were conducted for the top models, and a significant test indicates a deviation of the model simulated summary statistics from the observed summary statistics.

Parameter estimates (Table 6) using approximate Bayesian computation had large confidence intervals around most estimates, suggesting rather flat probability distributions. Comparing these estimates to those obtained from the allele frequency spectrum (Table 7) revealed that absolute values for estimates of population size were often an order of magnitude larger and gene flow rates were an order of magnitude smaller under the allele frequency spectrum. However, for a few population size estimates, the parameter estimates from the allele frequency spectrum fell below the bootstrapped confidence intervals, suggesting that these results may also be imprecise. Nevertheless, estimates of effective population size are not consistent with any strong population bottlenecks. Both approaches indicated limited rates of gene flow (*m* < 0.03) among populations across species, and rates were similar among long‐winged and short‐winged species across methods. While the estimated population size did not vary systematically with elevation in most cases, the *A. clavatus* upslope model and the *M. sanguinipes* source‐sink model suggested larger population sizes at lower elevation. Two species had single populations with reduced population sizes, a mid‐elevation site for *A. clavatus*, and the highest elevation site for *C. pellucida*.

**Table 6:** Median parameter estimates for effective size (*N*_e_) and migration rate (*m*), with 95% confidence intervals (in parentheses) based on Approximate Bayesian computation using the neural net function

	*Aeropedellus clavatus*	*Melanoplus boulderensis*	*Melanoplus sanguinipes*			*Camnula pellucida*
	Stepping stone	Stepping stone	Stepping stone	Downslope	Source‐sink	Upslope
*N*_e_ 1,577 m	N/A	N/A	1,433 (CI: 221–9,069)	1,395 (CI: 221–9,079)	1,401 (CI: 220–9,066)	1,232 (CI: 144–8,771)
*N*_e_ 2,195 m	1,413 (CI: 220–9,070)	1,397 (CI: 220–9,076)	1,423 (CI: 221–9,042)	1,437 (CI: 220–9,055)	1,386 (CI: 220–9,046)	1,533 (CI: 290–7,390)
*N*_e_ 2,591 m	1,425 (CI: 221–9,082)	1,411 (CI: 219–9,062)	1,410 (CI: 220–9,062)	1,408 (CI: 221–9,067)	19 (CI: 3–123)	3,349 (CI: 615–18,027)
*N*_e_ 3,048 m	1,395 (CI: 220–9,012)	1,414 (CI: 220–9,046)	2,130 (CI: 188–7,725)	1,387 (CI: 219–9,056)	1,415 (CI: 221–9,080)	N/A
*N*_e_ 3,515 m	N/A	1,419 (CI: 220–9,054)	1,394 (CI: 220–9,078)	1,354 (CI: 214–8,766)	1,394 (CI: 219–9,015)	179 (CI: 25–1,487)
*m*	0.025 (CI: 0.002–0.049)	0.026 (CI: 0.002–0.049)	0.025 (CI: 0.002–0.049)	0.030 (CI: 0.003–0.058)	0.026 (CI: 0.002–0.049)	0.0076 (CI: 0.004–0.011)

**Table 7:** Parameter estimates for effective size (*N*_e_) and migration rate (*m*), with bootstrap confidence intervals (in parentheses) based on the joint allele frequency spectrum

	*Aeropedellus clavatus*	*Melanoplus boulderensis*	*Melanoplus sanguinipes*		*Camnula pellucida*
	Upslope	Stepping stone	Downslope	Source‐sink	Stepping stone
*N*_e_ 1,577 m	16,040 (CI: 15,284–16,887)	N/A	9,083 (CI: 8,469–11,582)	15,887 (CI: 15,009–16,550)	N/A
*N*_e_ 2,195 m	344 (CI: 261–386)	15,157 (CI: 16,483–21,626)	2,997 (CI: 3,585–4,781)	4,481 (CI: 3,670–5,195)	16,674 (CI: 16,567–18,154)
*N*_e_ 2,591 m	10,820 (CI: 12,550–18,676)	16,040 (CI: 16,687–23,278)	3,060 (CI: 3,329–4,014)	3,157 (CI: 2,894–3,728)	1,204 (CI: 923–1,388)
*N*_e_ 3,048 m	N/A	15,870 (CI: 15,550–23,839)	4,679 (CI: 3,520–5,316)	4,482 (CI: 2,649–4,456)	2,992 (CI: 2,349–3,475)
*N*_e_ 3,515 m	767 (CI: 622–925)	15,279 (CI: 16,482–21,890)	15,660 (CI: 14,556–16,233)	801 (CI: 799–1,012)	N/A
*m*	0.005 (CI: 0.004–0.005)	0.001 (CI: 0.0002–0.0007)	0.001 (CI: 0.001–0.001)	0.001 (CI: 0.001–0.001)	0.001 (CI: 0.001–0.001)

### 3.4 Phenology and phenological overlap

**Figure 5:**
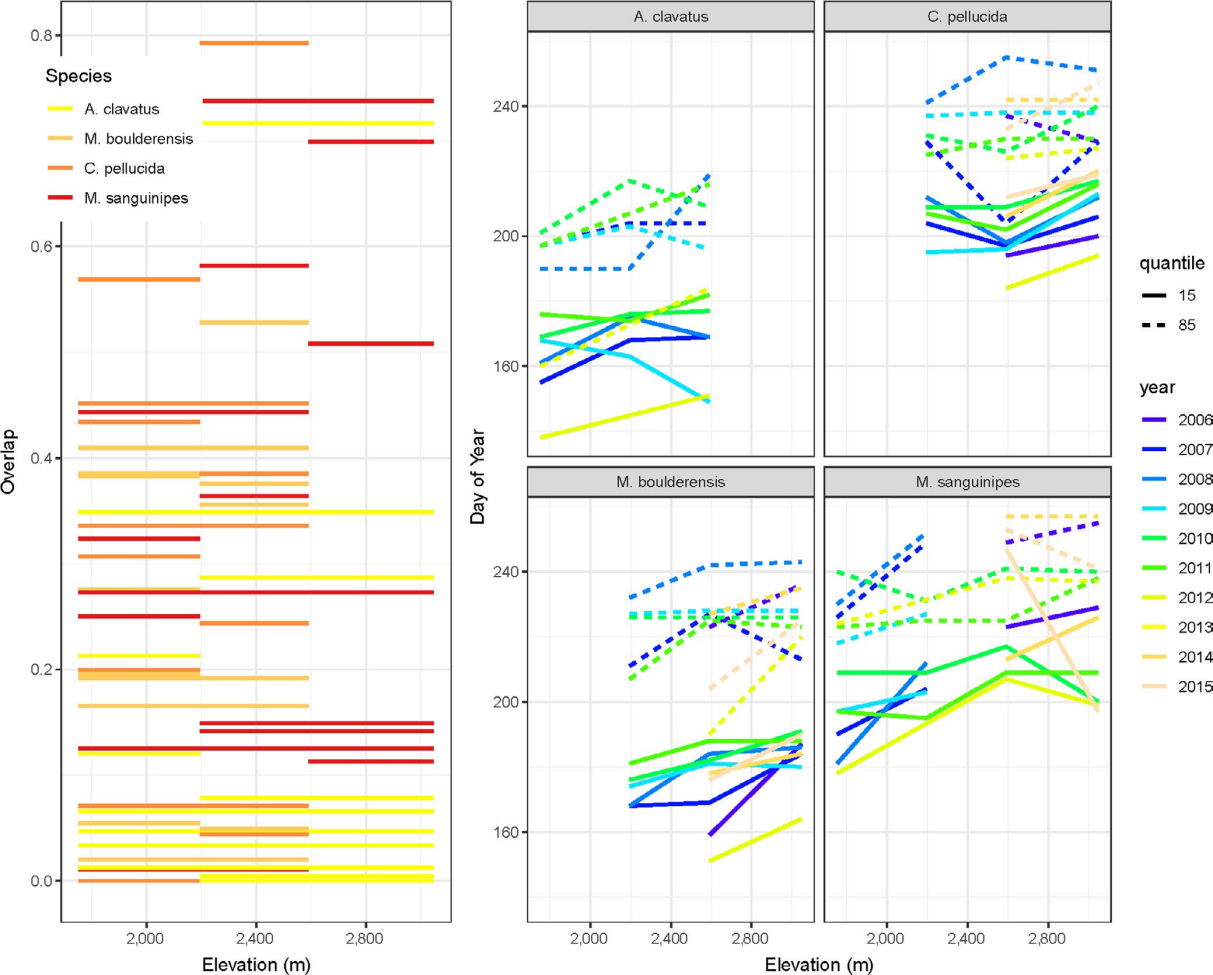
The extent of phenological overlap between populations varies among species and with the elevational separation of populations (left). We quantify phenological overlap using Pianka's index, which accounts for the abundance of individuals and ranges from 0 to 1 (no to complete overlap). Lines indicate the phenological overlap (*y*‐axis value) for the populations (elevations) connected by the line in a single survey year. We additionally examine phenological overlap as the shift in the phenology of the 15th and 85th seasonal percentile individual as a function of elevation (right)

Phenological overlap among sites, reflecting the potential for population mixing and gene flow, varies among years and along the elevation gradient (Figure 5 left panel). In an analysis of variance controlling for year, phenological overlap varies significantly among species (*F*_3,111_ = 4.52, *p* < .005) with overlap along the elevation gradient increasing in the following order: *A. clavatus*, *M. boulderensis*, *C. pellucida*, and *M. sanguinipes*. This order corresponds to the earlier season, short‐winged species exhibiting less overlap among sites (Figure 5 right panel). The appearance dates of the 15th and 85th percentile individuals increase with increasing elevation and vary significantly among species and years, but the slope of the relationships with elevation does not vary significantly among species (for the 15th percentiles, elevation: *F*_1,75_ = 62.7, *p* < .005, species: *F*_3,75_ = 110.0, *p* < .005, year: *F*_9,75_ = 6.3, *p* < .005, elevation × species: *F*_3,75_ = 0.9, *p* = .5; for the 85th percentiles, elevation: *F*_1,75_ = 70.8, *p* < .005, species: *F*_3,75_ = 61.4, *p* < .005, year: *F*_9,75_ = 5.6, *p* < .005, elevation × species: *F*_3,75_ = 0.8, *p* = .5;).

## 4 DISCUSSION

### 4.1 Local adaptation versus phenotypic plasticity in life‐history traits

We tested the hypothesis that dispersal ability would predict patterns of genomic and phenotypic differentiation in four ecologically divergent grasshopper species along an elevation transect in the Rocky Mountains. Relative to the long‐winged species, we expected the short‐winged species to exhibit stronger genetic differentiation, limited phenological overlap and gene flow, and a strong association between genetic divergence and phenotypic differentiation along the elevational cline. While our results for genomic divergence, gene flow, and phenology were supported, we found more equivocal evidence for associations between genetic and phenotypic variation that might be consistent with local adaptation.

First, genome‐wide genetic data confirm that all four species exhibit population genetic differentiation despite the short geographical distances among sites. That is, although populations along this elevation gradient are in close geographical proximity, their estimated gene flow rates are low, generally in a bidirectional stepping stone pattern. Consistent with our predictions, short‐winged species (*A. clavatus* and *M. boulderensis*) exhibit greater genetic differentiation (in pairwise *F*_ST_ and clustering results) than the long‐winged species (*C. pellucida* and *M. sanguinipes*), suggesting reduced dispersal ability. Phenological overlap among populations, an ecological measure of potential gene flow, reinforce these differences among short and long‐winged species.

Second, we examined whether genetic distance among populations could predict clinal differences in growth and reproduction “(life‐history traits)” and physiological traits of each species. Clinal divergence with limited gene flow can be consistent with local adaptation, as the strength of selection need not be exceptionally strong to maintain adaptive alleles (Kawecki & Ebert, 2004). However, we do not see a clear statistical association between genetic distance and phenotypic divergence. In the short‐winged species, statistically well‐supported differences in life‐history and physiological phenotypes along this elevational gradient (Buckley & Nufio, 2014; Buckley et al., 2014; Levy & Nufio, 2015) are not statistically correlated with genetic divergence. While this may be a result of modelling such relationships assuming a linear relationship and constancy in the scaler relationships among variables, or perhaps simply because we have a limited number of sites to test for associations, it also remains plausible that phenotypic plasticity could be controlling phenotypic trait distributions. For example, in the long‐winged species in our study, there is a lack of statistical associations between phenotype and environmental, elevational and even geographical predictor variables. Furthermore, experimental data demonstrate phenotypic plasticity for hopping performance in *M. sanguinipes*, as well as developmental plasticity due to rearing temperature (Buckley & Nufio, 2014). While controlled experiments have not been done to demonstrate plasticity in *C. pellucida*, it also exhibits elevation clines in the temperature dependence of hopping performance (Buckley & Nufio, 2014; Buckley et al., 2014).

Other studies with grasshoppers have found strong genetic structure at a small spatial scale while phenotypic patterns are decoupled from such genetic variation (Ortego et al., 2015; Tinnert & Forsman, 2017). Future efforts to understand phenotypic patterns in our focal species will need to employ reciprocal transplant experiments to directly test predictions about local adaptation and plasticity and to determine if fitness trade‐offs persist despite gene flow across these small spatial scales (Hereford, 2009). We currently lack relative fitness data to confirm that the phenotypic clines are adaptive. However, Levy and Nufio (2015) documented differential clines in both reproductive potential and realized reproduction for these four species. The number of functional ovarioles (egg‐producing tubules) increases with elevation for *M. boulderensis*, remains constant for *A. clavatus*, and decreases for the long‐winged species *C. pellucida* and *M. sanguinipes*. Thus, preliminary data at least suggest that local adaptation may be present in the short‐winged species, as reproductive provisioning varies while overall fitness remains high across the elevation gradient.

### 4.2 Patterns of genetic divergence and modelling gene flow along elevational gradients

Across environmental gradients, the amount and direction of gene flow between populations influence the evolutionary processes controlling phenotypic variation. Gene flow is known to facilitate adaptation by increasing the genetic diversity upon which selection can act, but it is more likely to reduce divergent selection on an ecological gradient as it allows an influx of maladaptive alleles to populations along a cline (Lenormand, 2002; for an interesting exception, see Sexton, Strauss, & Rice, 2011). Principle component and clustering analyses show that all four grasshopper species have genetically distinct populations along this 2,000 m elevation cline. For *A. clavatus*, genetic structure is consistent with isolation by distance. Although Mantel tests of isolation by distance are nonsignificant in *C. pellucida*, gradual genetic differentiation (*F*_ST_) across distance and a horseshoe‐shaped pattern in PC1 and PC2 (Frichot, Schoville, Bouchard, & François, 2012) are common patterns caused by isolation by distance. For *M. boulderensis*, populations' exhibit gradual genetic differentiation until the highest elevation, where that population is strongly divergent genetically. Finally, while middle elevation populations are structured in *M. sanguinipes*, the lowest elevation and highest elevation populations overlap strongly, suggesting a source‐sink dynamic. These observations support the contention of the original surveyor (Alexander, 1964) that *M. sanguinipes* is an accidental disperser into alpine sites. Note, none of these patterns suggest secondary contact following a long period of isolation, given that genetic divergence is relatively modest for all species (*F*_ST_ < 0.085).

We employed Approximate Bayesian computation to estimate the best fitting models of gene flow among populations along the elevational transect. The island model with equal gene flow among all populations was strongly rejected in all species. For *A. clavatus*, the upslope model was favored by both ABC model fitting approaches. We found discrepancies in the rejection and neural network estimates for a best fitting model in the other three species. Using neural networks, the stepping stone model of bidirectional gene flow was favored for *M. boulderensis*, *M. sanguinipes*, and *C. pellucida*, while the downslope model retained marginal support in all three species. In *M. sanguinipes*, a source‐sink model*,* whereby all populations contribute to the high‐elevation population, also showed marginal support. The neural network approach is thought to perform better when the number of summary statistics is large and to provide more accurate results (Blum & François, 2010; Csilléry, Blum, Gaggiotti, & François, 2010). However, the result of the modelling exercise suggests considerable uncertainty remains, particularly in parameter estimates. It is clear that gene flow among populations is present, although limited along the elevational transect, but the direction and rate of gene flow may not be accurate. We acknowledge that our models relied on simplistic assumptions (e.g., a single rate of gene flow across all populations) that likely impact these parameter estimates. However, there is inherent difficulty in estimating gene flow (Marko & Hart, 2011), especially using approaches reliant on summary statistics. We, therefore, conducted additional simulations to estimate gene flow from the joint allele frequency spectrum of each species, but bootstrapping suggested parameter estimates remained imprecise. Uncertainty in gene flow estimates may be a consequence of insufficient sampling in our study system, or simply a consequence of the limits of the above methods in inferring demographic parameters in some evolutionary scenarios (Cayuela et al., 2018).

### 4.3 Ecological determinants of gene flow

The amount and direction of gene flow are determined not only by dispersal capacity and population size, but also the degree of nonrandom mating. Variation in, for example, activity and reproductive timing could limit gene flow between populations in different environments (Sexton, Hangartner, & Hoffmann, 2014). Later phenology at higher elevations may contribute to the prevalence of upslope migration. Consistent will limited phenological overlap reducing gene flow, the alpine populations of *A. clavatus* and *M. boulderensis* were found to be particularly genetically isolated and also to exhibit phenotypic differentiation. In contrast, the steepness of the elevational cline in phenology varies among years for *M. sanguinipes*, which may allow gene flow to override genetic differentiation. The short‐winged species, *A. clavatus* and *M. boulderensis*, are active early in the season and are also less dispersive among the grasshopper species in our study. Thus, low phenological overlap may act to reduce gene flow and contribute to genetic differentiation. Early seasonal species are often adapted for rapid development, which can lead them to capitalize on warming and exhibit the most pronounced phenological shifts (Buckley et al., 2015). Phenological timing may be further constrained by snowmelt timing at high‐elevation sites, leading to low phenological overlap in heavy‐snow years. Phenological differences are frequently invoked as an agent of reduced gene flow in plants (Franks & Weis, 2009), but the genetic implications of phenological differences have been less studied for insects (Mopper, 2005). In one example, upslope migration was found to result from butterflies emigrating away from senescing host plants at low elevation (Peterson, 1997). Other studies have found reduced genetic variation and emerging genetic structure in high‐elevation insect populations (Jackson et al., 2018; Polato et al., 2017), although it is unclear if this pattern is due to phenological mismatch.

Gene flow patterns could also be driven by how wind direction facilitates dispersal. In particular, the genetic clustering of *M. sanguinipes* populations from the elevational extremes suggests an important role of long distance, passive dispersal by the wind. During summer in the mountains, the prevailing direction of airflow near the ground is upward during the day and downward at night. This air movement, due to radiation and the gradient of air density, flows counter to the prevailing winds in our study region (Alexander, 1964). Reduced grasshopper activity at night leads to most movement occurring predominately upslope during the day. Recent survey records suggest the greatest incidence of transient individuals occurs during periods of high temperature and low wind speeds in the upslope direction (C. Nufio, unpublished data; during high winds, grasshoppers are less likely to take flight). As such, more migrants of *M. sanguinipes* may reach the alpine from the lowest elevation site than the mid‐elevation sites due to higher population densities at low elevation. For *M. sanguinipes*, surveys found only adults (no juveniles) at upper elevation sites, suggesting a lack of reproduction or limited recruitment in these populations. This is reinforced by observed declines in reproductive potential (proportion of ovarioles that become functional) with elevation for *M. sanguinipes* (Levy & Nufio, 2015) suggesting that low elevation migrants are poorly suited to the alpine. Populations composed of transient individuals may therefore represent a sink population, rather than a stable local population (Gaggiotti, 1996).

Climate change could quickly alter genetic structure in these species. Dispersal may increase as warm days increase the upward flow of air near the ground. Population differentiation, as observed in the alpine populations of *A. clavatus* and *M. boulderensis*, could subsequently erode and upslope flow of warm‐adapted alleles could facilitate adaptive responses to climate change. However, climate change could also decrease phenological overlap among populations and exacerbate population differentiation. High‐elevation populations might then contract and experience greater fragmentation (Rubidge et al., 2012). An example of environmental conditions influencing phenological overlap is provided by plant responses to drought: drought alters phenology more for wet‐adapted plants than dry‐adapted plants, increasing phenological overlap (Franks & Weis, 2008). Because grasshopper populations differ in the temperature dependence of their developmental rates (Buckley et al., 2015), and previous documentation of phenological advancements show an association with climate warming (Nufio & Buckley, 2019), we predict that phenological timing will move earlier in the year and will be exacerbated by changes to snowmelt and plant phenology patterns along the gradient. Interspecific phenological mismatch within sites has been extensively studied as a driver of altered species interactions (Visser & Both, 2005), but intraspecific phenological mismatch may be equally important to climate change outcomes as it reduces gene flow among sites.

## CONFLICT OF INTEREST

None declared.

## AUTHOR CONTRIBUTIONS

All authors assisted in paper writing. L.B.B., R.A.S., and S.D.S. conceived the study and L.B.B., C.R.N, and R.A.S. collected specimens. R.A.S. and S.D.S. conducted the genetic data collection and analyses, C.R.N. conducted the phenological surveys.

## Data Availability

Raw Illumina reads were deposited in the National Center for Biotechnology Information Short Read Archive (accession nos. SRR9216863–SRR9216866) under BioProject PRJNA547722. Additional supplementary tables and figures, as well the barcode, sample identifier and population identifier for demultiplexing Illumina short read data, and custom R scripts and input files for FASTSIMCOAL2 simulations, are available on DRYAD (https://doi.org/10.5061/dryad.w3r2280m3).
