# Supplementary material for: Do different rates of gene flow underlie variation in phenotypic and phenological clines in a montane grasshopper community?
Source: Ecol Evol. 2019 Dec 30;10(2):980–97. doi: 10.1002/ece3.5961 (PMC6988534; doi:10.1002/ece3.5961)
Supplement: Supplementary file 1 [file ECE3-10-980-s001.docx]

**Supplemental File 1**

**Do different rates of gene flow underlie variation in phenotypic and phenological clines in a montane grasshopper community?**

Rachel A. Slatyer, Sean D. Schoville^,^ César R. Nufio^,^and Lauren B. Buckley

**Table S1**. Confusion matrices for *Camnula pellucida*, showing model discrimination among simulated data at varying levels of tolerance for the rejection and neural network methods*.*

|  | **Rejection method** | | | |  | **Neural network** | | | |
| --- | --- | --- | --- | --- | --- | --- | --- | --- | --- |
| Tolerance = 0.001 | am | dn | ss | up |  | am | dn | ss | up |
| am | 87 | 0 | 7 | 4 |  | 0.73 | 0.03 | 0.19 | 0.05 |
| dn | 2 | 97 | 1 | 0 |  | 0.04 | 0.88 | 0.04 | 0.03 |
| ss | 11 | 0 | 89 | 0 |  | 0.23 | 0.02 | 0.72 | 0.03 |
| up | 1 | 2 | 5 | 92 |  | 0.04 | 0.03 | 0.06 | 0.87 |
|  |  |  |  |  |  |  |  |  |  |
| Tolerance = 0.005 | am | dn | ss | up |  | am | dn | ss | up |
| am | 93 | 0 | 4 | 3 |  | 0.71 | 0.04 | 0.19 | 0.06 |
| dn | 2 | 98 | 0 | 0 |  | 0.06 | 0.83 | 0.06 | 0.05 |
| ss | 13 | 0 | 87 | 0 |  | 0.23 | 0.03 | 0.70 | 0.04 |
| up | 2 | 2 | 3 | 93 |  | 0.05 | 0.04 | 0.07 | 0.84 |
|  |  |  |  |  |  |  |  |  |  |
| Tolerance = 0.01 | am | dn | ss | up |  | am | dn | ss | up |
| am | 96 | 1 | 1 | 2 |  | 0.65 | 0.06 | 0.23 | 0.07 |
| dn | 2 | 98 | 0 | 0 |  | 0.08 | 0.79 | 0.08 | 0.06 |
| ss | 19 | 0 | 81 | 0 |  | 0.27 | 0.05 | 0.62 | 0.06 |
| up | 4 | 2 | 4 | 90 |  | 0.08 | 0.06 | 0.09 | 0.78 |

**Table S2**. Confusion matrices for *Melanoplus boulderensis,* showing model discrimination among simulated data at varying levels of tolerance for the rejection and neural network methods*.*

|  | **Rejection method** | | | |  | **Neural network** | | | |
| --- | --- | --- | --- | --- | --- | --- | --- | --- | --- |
| Tolerance = 0.001 | am | dn | ss | up |  | am | dn | ss | up |
| am | 85 | 1 | 13 | 1 |  | 0.84 | 0.01 | 0.13 | 0.01 |
| dn | 5 | 90 | 2 | 3 |  | 0.01 | 0.94 | 0.03 | 0.02 |
| ss | 21 | 1 | 78 | 0 |  | 0.09 | 0.03 | 0.83 | 0.04 |
| up | 5 | 0 | 1 | 94 |  | 0.03 | 0.03 | 0.04 | 0.91 |
|  |  |  |  |  |  |  |  |  |  |
| Tolerance = 0.005 | am | dn | ss | up |  | am | dn | ss | up |
| am | 83 | 2 | 14 | 1 |  | 0.83 | 0.02 | 0.13 | 0.02 |
| dn | 10 | 81 | 5 | 4 |  | 0.02 | 0.90 | 0.04 | 0.04 |
| ss | 28 | 2 | 69 | 1 |  | 0.13 | 0.04 | 0.78 | 0.05 |
| up | 9 | 0 | 4 | 87 |  | 0.05 | 0.04 | 0.05 | 0.86 |
|  |  |  |  |  |  |  |  |  |  |
| Tolerance = 0.01 | am | dn | ss | up |  | am | dn | ss | up |
| am | 85 | 2 | 11 | 2 |  | 0.79 | 0.03 | 0.14 | 0.03 |
| dn | 14 | 76 | 5 | 5 |  | 0.04 | 0.84 | 0.06 | 0.07 |
| ss | 31 | 3 | 64 | 2 |  | 0.16 | 0.06 | 0.72 | 0.06 |
| up | 16 | 0 | 5 | 79 |  | 0.06 | 0.08 | 0.08 | 0.79 |

**Table S3**. Confusion matrices for *Melanoplus sanguinipes,* showing model discrimination among simulated data at varying levels of tolerance for the rejection and neural network methods*.*

|  | **Rejection method** | | | |  | **Neural network** | | | | | | |
| --- | --- | --- | --- | --- | --- | --- | --- | --- | --- | --- | --- | --- |
| Tolerance = 0.001 | am | dn | sk | ss | up |  | am | dn | sk | ss | up |  |
| am | 100 | 0 | 0 | 0 | 0 |  | 0.92 | 0.01 | 0.01 | 0.05 | 0.01 |  |
| sk | 0 | 99 | 0 | 0 | 1 |  | 0.01 | 0.78 | 0.01 | 0.01 | 0.19 |  |
| dn | 0 | 0 | 100 | 0 | 0 |  | 0.01 | 0.01 | 0.92 | 0.03 | 0.02 |  |
| ss | 0 | 0 | 0 | 99 | 1 |  | 0.03 | 0.01 | 0.03 | 0.92 | 0.02 |  |
| up | 0 | 7 | 1 | 0 | 92 |  | 0.01 | 0.17 | 0.002 | 0.03 | 0.78 |  |
|  |  |  |  |  |  |  |  |  |  |  |  |  |
| Tolerance = 0.005 | am | dn | Sk | ss | up |  | am | dn | sk | ss | up |  |
| am | 96 | 0 | 0 | 4 | 0 |  | 0.90 | 0.01 | 0.03 | 0.07 | 0.01 |  |
| sk | 0 | 97 | 0 | 0 | 3 |  | 0.01 | 0.69 | 0.03 | 0.01 | 0.26 |  |
| dn | 0 | 1 | 98 | 1 | 0 |  | 0.01 | 0.04 | 0.87 | 0.04 | 0.04 |  |
| ss | 0 | 0 | 0 | 100 | 0 |  | 0.05 | 0.01 | 0.03 | 0.89 | 0.02 |  |
| up | 0 | 7 | 0 | 1 | 92 |  | 0.01 | 0.22 | 0.05 | 0.04 | 0.69 |  |

**Table S4**. Confusion matrices for *Aeropedellus clavatus*, showing model discrimination among simulated data at varying levels of tolerance for the rejection and neural network methods*.*

|  | **Rejection method** | | | |  | **Neural network** | | | |
| --- | --- | --- | --- | --- | --- | --- | --- | --- | --- |
| Tolerance = 0.001 | am | dn | ss | up |  | am | dn | ss | up |
| am | 100 | 0 | 0 | 0 |  | 0.93 | 0.00 | 0.07 | 0.00 |
| dn | 1 | 99 | 0 | 0 |  | 0.00 | 0.98 | 0.00 | 0.02 |
| ss | 3 | 0 | 97 | 0 |  | 0.09 | 0.00 | 0.89 | 0.01 |
| up | 0 | 0 | 0 | 100 |  | 0.00 | 0.01 | 0.01 | 0.98 |
|  |  |  |  |  |  |  |  |  |  |
| Tolerance = 0.005 | am | dn | ss | up |  | am | dn | ss | up |
| am | 100 | 0 | 0 | 0 |  | 0.94 | 0.00 | 0.06 | 0.00 |
| dn | 1 | 97 | 1 | 1 |  | 0.00 | 0.94 | 0.02 | 0.05 |
| ss | 10 | 0 | 30 | 0 |  | 0.11 | 0.01 | 0.87 | 0.01 |
| up | 1 | 1 | 1 | 97 |  | 0.01 | 0.03 | 0.02 | 0.94 |
|  |  |  |  |  |  |  |  |  |  |
| Tolerance = 0.01 | am | dn | ss | up |  | am | dn | ss | up |
| am | 100 | 0 | 0 | 0 |  | 0.94 | 0.00 | 0.06 | 0.00 |
| dn | 1 | 96 | 2 | 1 |  | 0.00 | 0.91 | 0.03 | 0.06 |
| ss | 12 | 0 | 88 | 0 |  | 0.09 | 0.01 | 0.87 | 0.01 |
| up | 2 | 2 | 1 | 95 |  | 0.01 | 0.05 | 0.04 | 0.90 |


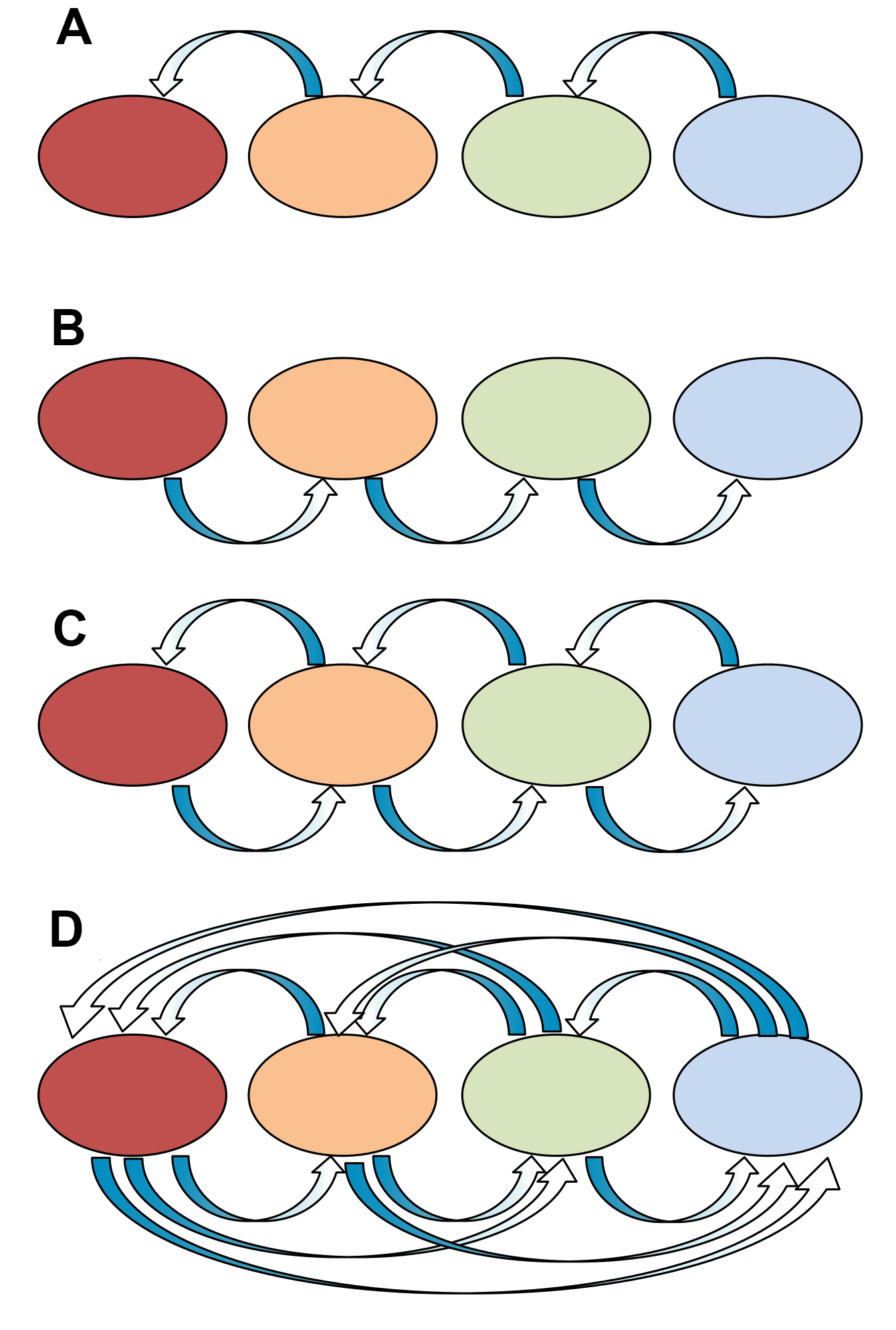


**Figure S1**. Spatially explicit demographic models used to simulate synthetic genetic datasets for Approximate Bayesian computation analysis. A) Downslope stepping stone model, B) upslope stepping stone model, C) symmetric stepping stone model, and D) an island model, with equal and constant gene flow among all populations.


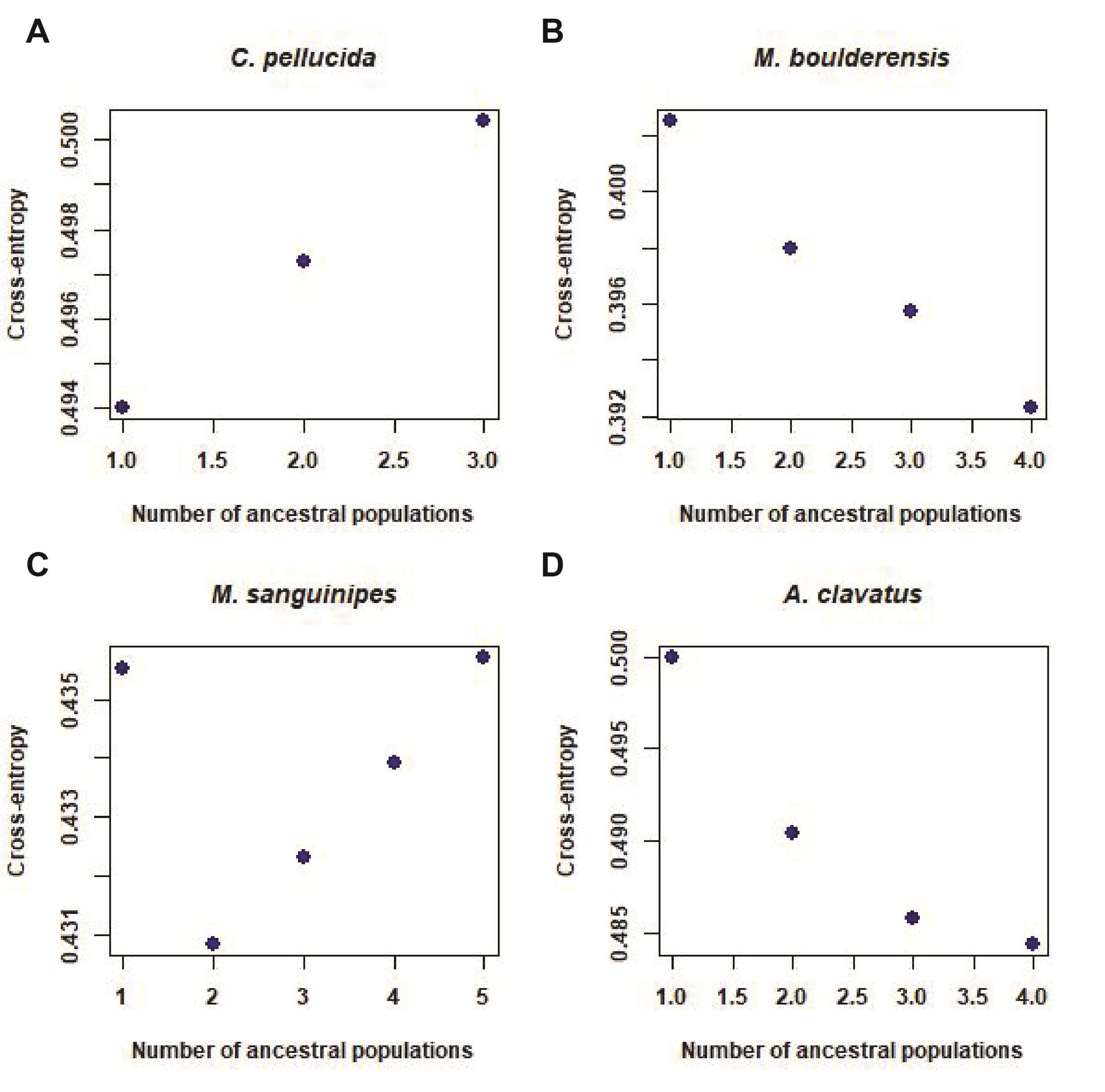


**Figure S2**. Cross entropy values for each value of *K* ancestral populations in the clustering algorithm sNMF, for (a) *Camnula pellucida*, (b) *Melanoplus boulderensis*, (c) *Melanoplus sanguinipes* and *D) Aeropedellus clavatus*.


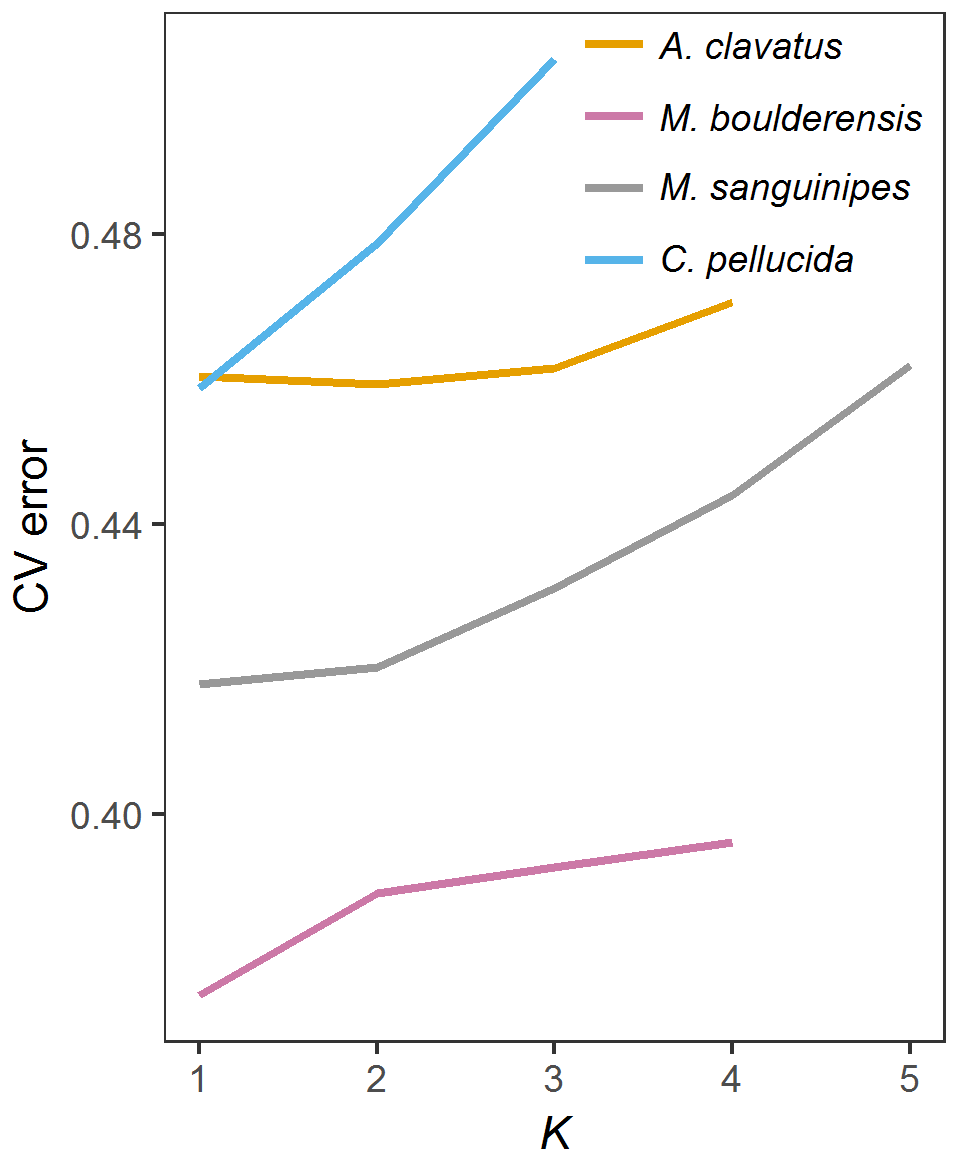


**Figure S3**. Cross validation error for each value of *K* ancestral populations in the clustering algorithm ADMIXTURE, for the species *Camnula pellucida*, *Melanoplus boulderensis*, *Melanoplus sanguinipes* and *Aeropedellus clavatus*.


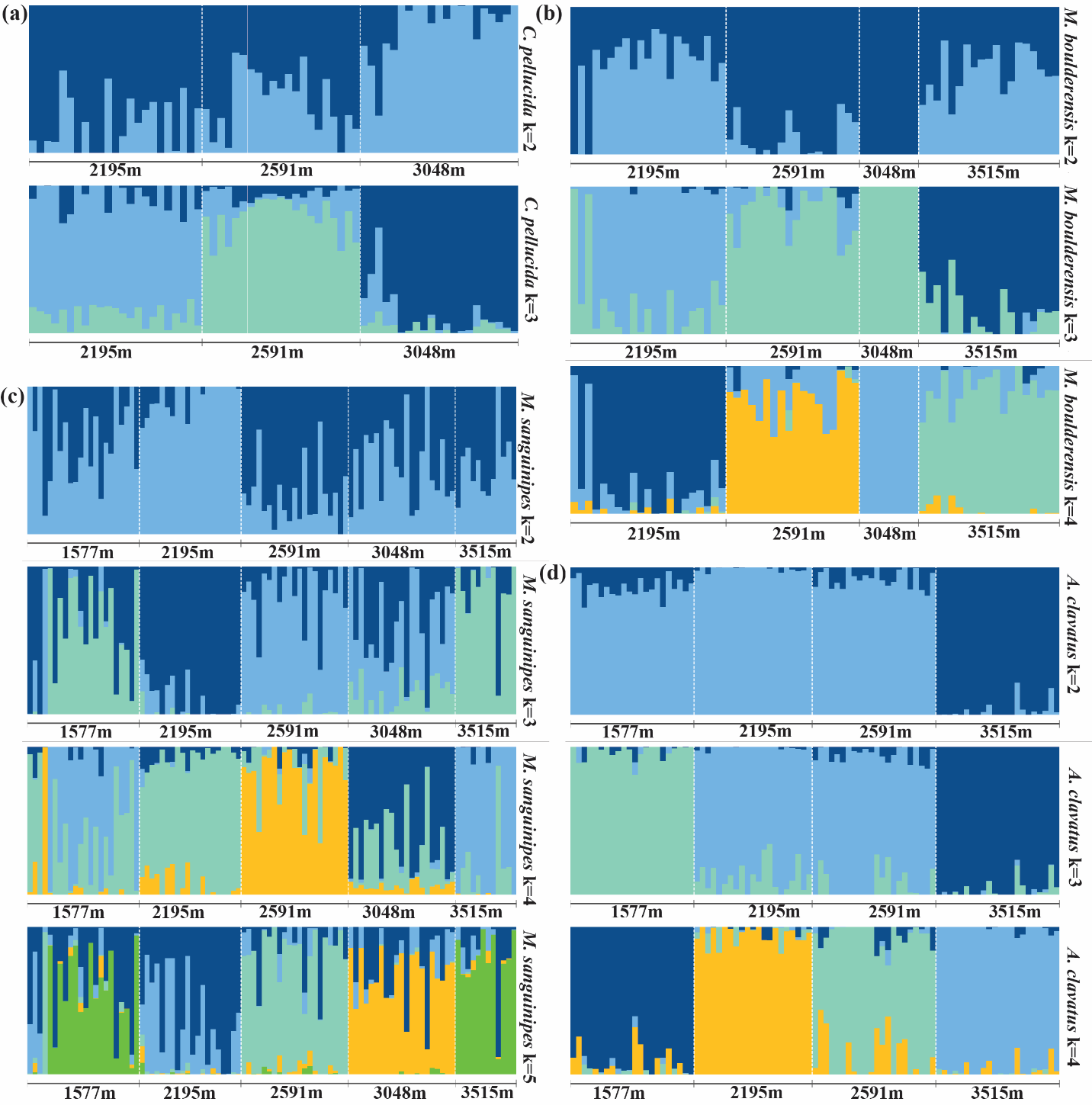


**Figure S4**. Ancestry proportions for (a) *Camnula pellucida*, (b) *Melanoplus boulderensis*, (c) *Melanoplus sanguinipes* and *D) Aeropedellus clavatus* modelled for varying levels of *K* ancestral populations (*K* level noted on side of graph), each generated from 10 independent runs using sNMF. Each bar is an individual and ancestry proportions represent the estimated proportion of an individual’s genome originating from each ancestral population (represented by different colours).

**
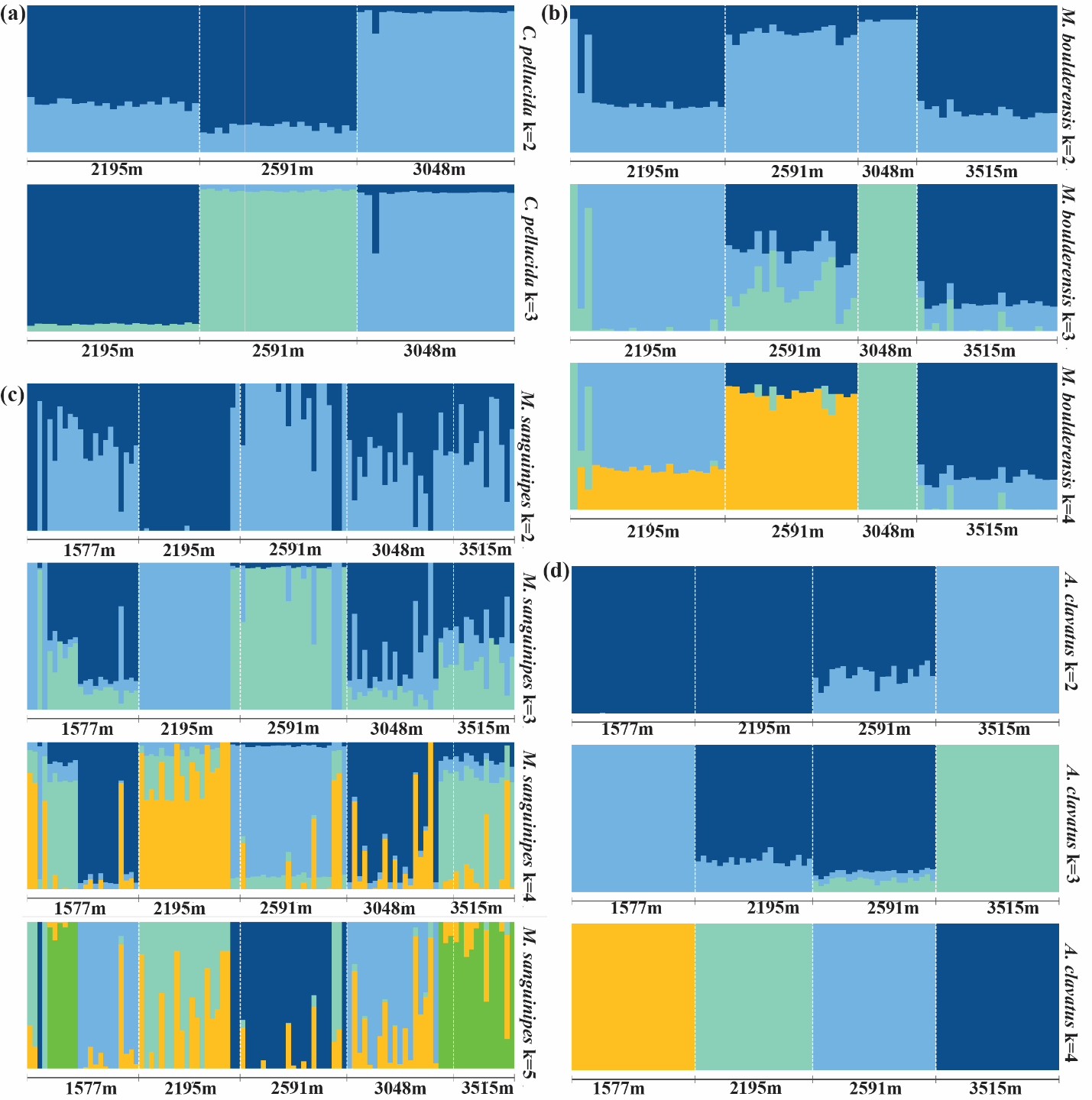
**

**Figure S5**. Ancestry proportions for (a) *Camnula pellucida*, (b) *Melanoplus boulderensis*, (c) *Melanoplus sanguinipes* and *D) Aeropedellus clavatus* modelled for varying levels of *K* ancestral populations (*K* level noted on side of graph), each generated from 10 independent runs using ADMIXTURE. Each bar is an individual and ancestry proportions represent the estimated proportion of an individual’s genome originating from each ancestral population (represented by different colours).
